# Establishment of reference intervals of thyroid-related hormones for adults with normal liver function in Zhejiang Province by indirect method

**DOI:** 10.3389/fendo.2024.1441090

**Published:** 2024-09-10

**Authors:** Xiying Huang, Xufeng Yang

**Affiliations:** Department of Lab Medicine, The Second Affiliated Hospital Zhejiang University School of Medicine, Hangzhou, Zhejiang, China

**Keywords:** 129, establishment of reference intervals, Zhejiang Province, adults with normal liver function, thyroid-related hormones, indirect method

## Abstract

**Objective:**

Thyroid disorders are prevalently diagnosed yet face significant challenges in their accurate identification in China. Predominantly, the reference intervals (RIs) currently in use across Chinese medical facilities derive from company-provided data, lacking stringent scientific validation. This practice underscores the urgent necessity for establishing tailored RIs for thyroid-related hormones, specifically tailored to the coastal area populations. Such refined RIs are imperative for empowering clinicians with the precise tools needed for the accurate diagnosis of both overt and subclinical thyroid conditions.

**Methods:**

This investigation analyzed the medical histories of 6021 euthyroid individuals mainly from East coastal area of China between June 2019 and December 2020. The cohort comprised residents of coastal areas, focusing on extracting insights into the regional specificity of thyroid hormone levels. A thorough examination protocol was implemented, encompassing inquiries into thyroid health history, ultrasound screenings, palpations during thyroid surgery, detections of thyroid antibodies, and reviews of medication histories. Adherence to the CLSI C28-A3 guidelines facilitated the derivation of RIs for thyroid-related hormones, subsequently juxtaposed against those provided by commercial entities.

**Results:**

The study delineated the following gender- and age-specific RIs for Thyroid-Stimulating Hormone (TSH): for males under 50 years, 0.57-3.37; males over 50 years, 0.51-4.03; females under 50 years, 0.53-3.91; and females over 50 years, 0.63-4.31. Further analysis revealed the RIs for Free Thyroxine (FT4), Free Triiodothyronine (FT3), Total Thyroxine (TT4), and Total Triiodothyronine (TT3) amongst males and females, with notable distinctions observed between the two genders and across age brackets. These findings are in stark contrast to the standardized intervals provided by manufacturers, particularly highlighting differences in TT3 and FT3 levels between genders and a tendency for TSH levels to increase with age.

**Conclusion:**

This research successfully establishes refined RIs for thyroid-related hormones within the Chinese coastal area populations, taking into account critical demographic factors such as gender and age. These tailored RIs are anticipated to significantly enhance the diagnostic accuracy for thyroid diseases, addressing the previously noted discrepancies with manufacturer-provided data and underscoring the importance of regionally and demographically adjusted reference intervals in clinical practice.

## Introduction

The prevalence of thyroid dysfunction including primary asymptomatic thyroid disorders, in the coastal are of China, represents a significant public health challenge. This condition, often detected during routine health examinations, presents diagnostic difficulties due to the high incidence of subclinical manifestations. The Clinical and Laboratory Standards Institute (CLSI) (1, 2) underscores the complexity of accurately assessing thyroid health, acknowledging the nuanced nature of thyroid disorders and the imperative for refined diagnostic approaches.

The distribution of Thyroid-Stimulating Hormone (TSH) values among are not normally distributed, potentially as a result of preclinical thyroid infections (3). Epidemiological data suggest that 5%-15% of adults in China suffer from hypothyroidism, while 0.5%-2% of the population suffers from hyperthyroidism (4). Consequently, the evaluation of thyroid hormones has become a cornerstone in clinical practice for assessing thyroid function. Under such context, clinicians faces the critical task of delineating accurate and clinically relevant reference intervals (RIs) for thyroid-related hormones, an essential step towards improving the diagnosis and management of thyroid disorders.

To help physicians examine thyroid gland activity more precisely, the formulation of a regional typical RI for thyroid gland hormones was undertaken. The thyroid-related hormones we refer to here include TSH, FT4, FT3, TT4, and TT3. According to statistics, there are fewer than 100 hospitals in the world with regional reference intervals, and most hospitals still use Ris provided by the manufacturer. Based on existing reports, we are concerned that the Ris of thyroid-related hormones is affected by factors such as season, age, gender, different times of day, iodine levels, ethnicity, and differences in measurement methodology (5–8). Detection with different methodologies may have a greater impact on the thyroid-related hormone RIS (9). Therefore, we have more reason to establish an RIS based on the Zhejiang population and the hormone detection methodology of the second hospital affiliated with Zhejiang University.

This study adopts an indirect approach to establish robust and reliable RIs for thyroid-related hormones by leveraging a large dataset from the public health screening database of our institution. This method offers a practical, efficient, and cost-effective alternative to the traditional direct methods. Preliminary comparisons between the newly established RIs and those provided by manufacturers reveal significant discrepancies, highlighting the necessity for regionally adapted RIs in the effective clinical assessment of thyroid function within China’s coastal populations.

## Materials and methods

### Acquisition of information

**Figure 1:**
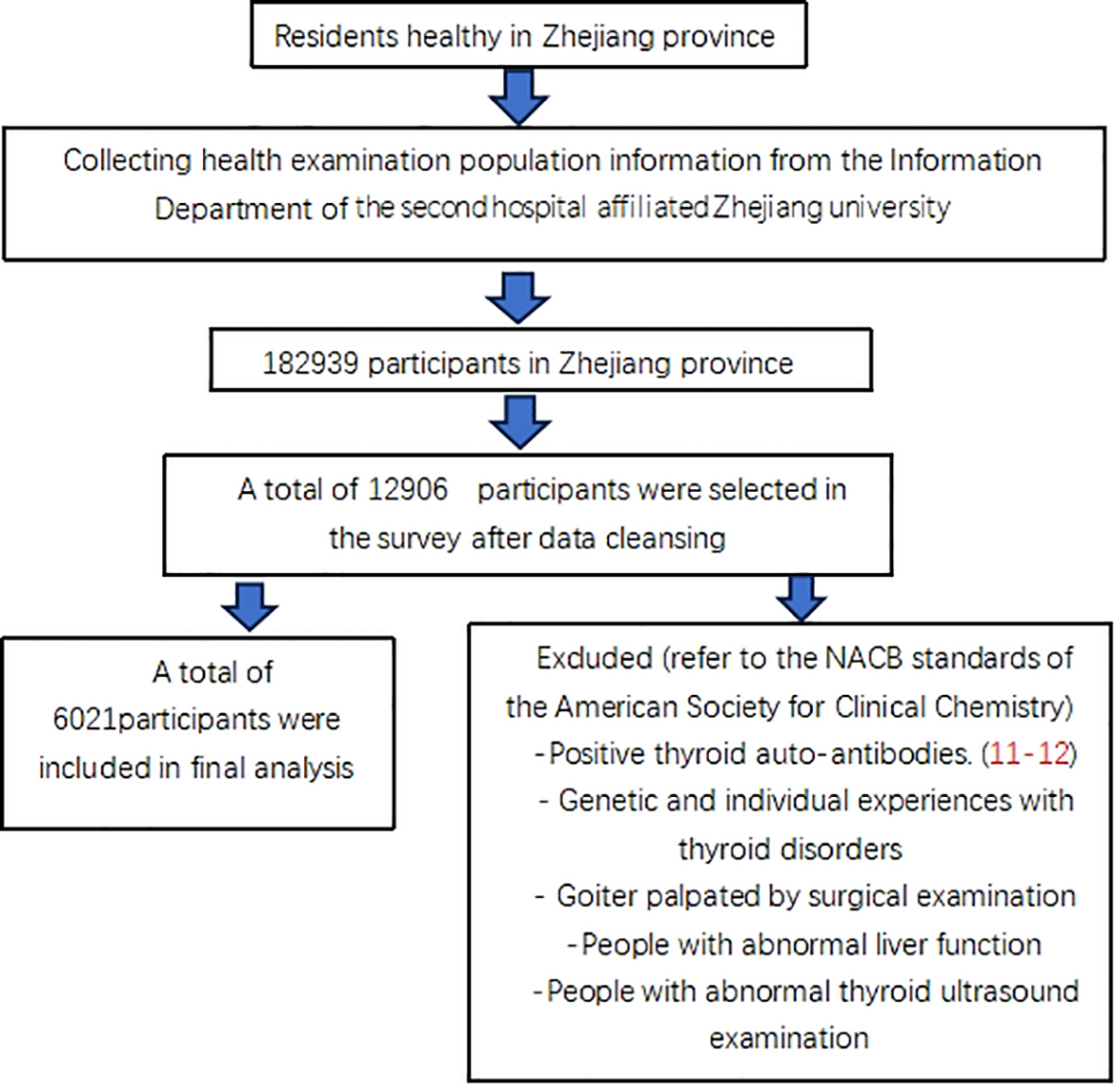
The accurate criteria for selection.

The data was taken from the Health Information System of the Second Affiliated Hospital of Zhejiang University from June to December (2019–20), pertaining to 182,939 healthy people in the Zhejiang Province of China. The information from6021 euthyroid people were ultimately included in this research (10). The additional anonymous analysis was performed, and **Figure 1** illustrates the accurate criteria for selection.

The hospital health screening populace dataset was the source of all of the information. During the ten minutes of quiet relaxation, the patient’s height, body mass index, and blood pressure were measured, and their blood was obtained on an empty stomach. The Body Mass Index (BMI) is calculated by dividing body weight in kilograms by the square of height in meters.). Following collection, the blood was spun in yellow-tipped tubes using an accelerating reagent for ten minutes at 3000 revolutions per minute. Five thyroid-related hormones were examined using an Abbott immunoassay analyzer equipped with matching reagents, calibrators, and quality control. The levels of ALT, Cr, TG, TC, and Glu were measured using the 5^th^ Olympus 5800 automated biochemical analyzer, which was equipped with quality controls, calibrators, and matching reagents.

The Ethics Review Committee at Zhejiang University’s Second Affiliated Hospital approved the current study.

The consent form is not required for this research because the findings are retrospective.

## Result

The participants’ demographic details are displayed in **Table 1**. The mean BMI was 23.1 and the average age was 40. While RIs for particular metrics differ for men and women, it’s important to keep in consideration that women are usually younger than men and exhibit a lower incidence of BMI, blood pressure (systolic and diastolic), ALT, Cr, white blood cell count (WBC), triglycerides (TG), low-density lipoprotein (LDL), and glucose (Glu).

**Figure 2:**
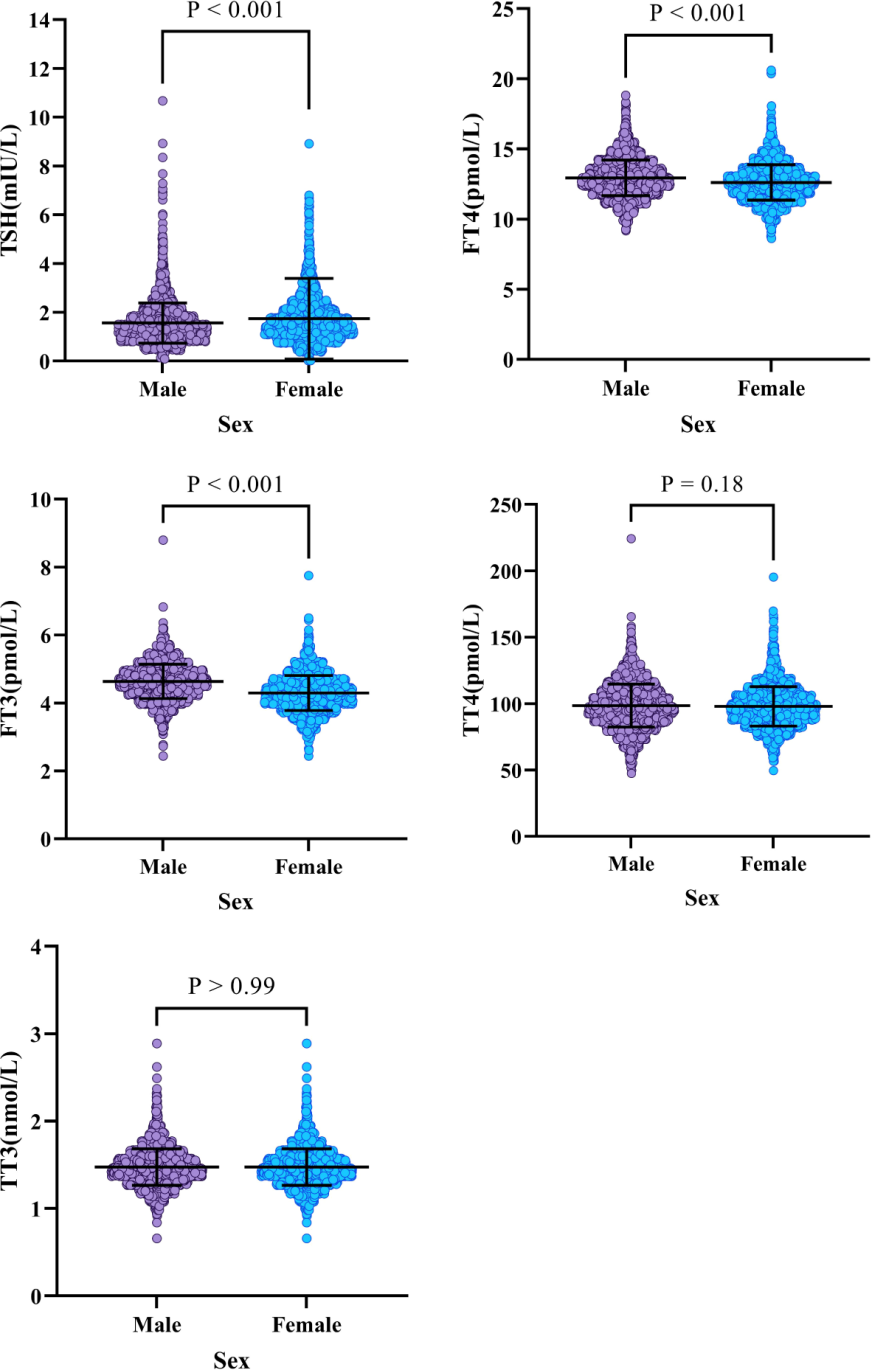
The levels of (1) Thyroid Stimulating Hormone (TSH), (2) Free Thyroxine (FT4), (3) Free Triiodothyronine (FT3), (4) Total Thyroxine (TT4), and (5) Total Triiodothyronine (TT3) were analyzed based on gender.

**Figure 3:**
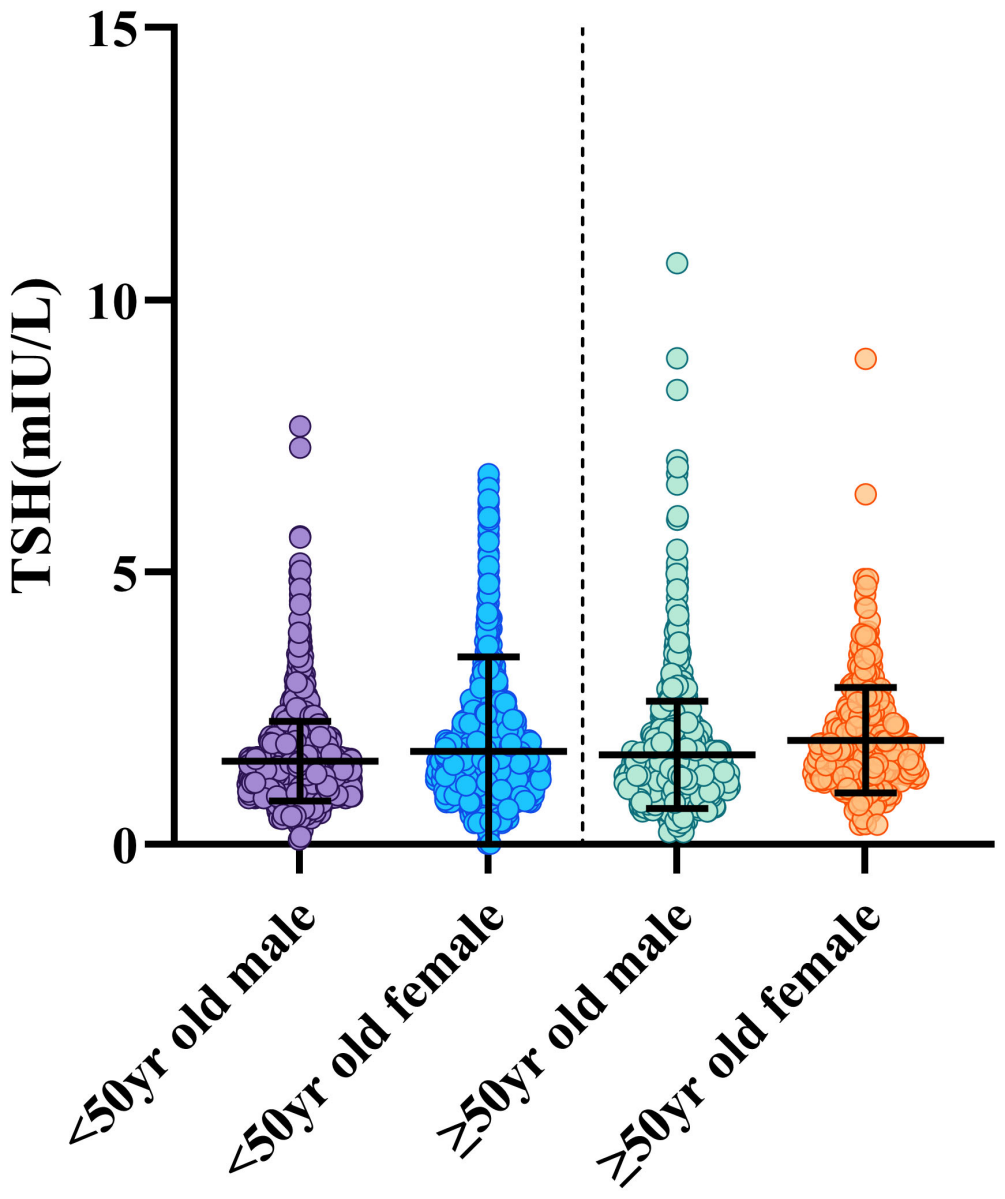
Participants were classified into two age groups: those over 50 and those under 50. The graphic displays the gender and age-related dynamic fluctuations in TSH. The data on men and females are displayed in different colour, including whiskers. The whiskers reach the 2.5th or 97.5th percentage, and the range across the 25th- 75th percentile. To show observed motion, averages are linked by lines and displayed as black lines inside the boxes.

1. The findings of a multiple regression study for thyroid-related hormones are presented in **Table 2**. In particular, gender-specific indices are significantly greater than other metrics. The distribution variations in thyroid hormones by gender are shown in **Figure 2**. Specifically, it showed that blood levels of Thyroid-Stimulating Hormone (TSH) increased in females compared to males (P<0.001). However, males indicated higher amounts of Free Triiodothyronine (FT3), and Free Thyroxine (FT4) (p<0.001). Gender did not have a significant impact on overallTotal Triiodothyronine (TT3) (p>0.99)and thyroid (TT4) values (p=0.18).
2. The exact RIs for thyroid-related hormones in normal adult Chinese coastal populations are listed in **Table 3**. Our results highlight the significance of developing sex-specific RIs for those five hormones. There is a strong association between TSH and age (P<0.001)). To further examine, we split the patients into 5 age-based categories: Group 1, ages 18–29 years old (N = 873); Group 2: 30-39 years old (N = 2100); Group 3: 40–49 years old (N = 1569); Group 4: 50–59 years old (N = 1015); Group 5 consisted of patients who were under 60 years old (N = 464). The TSH proportions of individuals under 50 and those over 50 varied, following subgroup analysis and pooling. From these results (**Figure 3**), we increased the thyroid-related hormone RIs for healthy people in China’s coastal areas.

**Table 1:** Basic demographic information of the healthy physical examination population.

Variables	Males	Females	Total
N	3579	2442	6021
Age (yr)	43 (25, 72)	36 (24, 61)	40 (24, 68)
BMI (kg/m^2^)	24 ± 2.9	21.6 ± 2.7	23.1 ± 3.1
SBP (mm Hg)	123 ± 16	111 ± 14	119 ± 16
DBP (mm Hg)	73 ± 11	66 ± 10	70 ± 11
WBC (10^9^/L)	6.1 ± 1.5	5.8 ± 1.4	6.0 ± 1.5
ALT (U/L)	21 (10, 38)	13 (7, 33)	17 (8, 38)
Cr (μmmol/L)	73 (55, 96)	52 (39, 68)	65 (42, 93)
TG (mmol/L)	1.3 (0.6, 4.3)	0.9 (0.5, 2.5)	1.1 (0.5, 3.7)
LDL (mmol/L)	2.7 (1.4, 4.1)	2.4 (1.3, 3.9)	2.5 (1.4, 4.1)
Glu (mmol/L)	5.0 (4.2, 7.4)	4.8 (4.1, 5.9)	4.9 (4.2, 6.9)

BMI, SBP, DBP, Cr, and Glu are displayed as the mean ± SD, however, the median (interquartile range) is employed for the other indexes.

BMI, body mass index; SBP, systolic blood pressure; DBP, diastolic blood pressure; Cr, creatinine; TG, triglycerides; Glu, glucose.

**Table 2:** Multiple linear regression.

	R^2^	GENDER	AGE	BMI	SBP
Thyroid-Stimulating Hormone (TSH)	0.016	0.226 0.037,**	0.004 0.001,**	0.011 0.006,0.109	0.002 0.001,0.118
Free Thyroxine (FT4)	0.063	-0.488 0.037,**	-0.023 0.001,**	-0.044 0.006,**	0.007 0.001,**
Free Triiodothyronine (FT3)	0.124	-0.320 0.015,**	-0.007 0.001,**	0.005 0.002,*	0.004 0.001,**
Total Thyroxine (TT4)	0.009	-1.239 0.466,**	-0.095 0.019,**	-0.355 0.074,**	0.062 0.014,**
Total Triiodothyronine (TT3)	0.07	-0.093 -0.006,*	-0.002 -0.002,**	0.003 0.003,**	0.001 0.001,**

R2, coefficient of determination; Standard Error, *P<0.05, **P<0.001, TSH, thyroid stimulating hormone; FT4, free thyroxine; FT3, free triiodothyronine; BMI, body mass index; SBP, systolic blood pressure: TT4, total thyroxine; TT3, total triiodothyronine

**Table 3:** The study’s RI for thyroid-associated hormones were contrasted with the manufacturer’s recommendations.

	GENDER	TSH	FT4	FT3	TT4	TT3
The current study	Male	<50yr old:1.38 (0.57,3.37)	12.87 (10.63,15.56)	4.64 (3.66,5.63)	97.80 (68.00,130.50)	1.57 (1.18,2.03)
		≥50yr old:1.42 (0.51,4.03)				
	Female	<50yr old:1.52 (0.53,3.91)	12.5 (10.39,15.31)	4.28 (3.34,5.32)	97.00 (72.32,131.66)	1.47 (1.09,1.92)
		≥50yr old:1.72 (0.63,4.31)				
Manufacturer	Total	1.45 (0.55,3.78)	12.75 (10.49,15.47)	4.49 (3.47,5.55)	97.40 (69.53,130.80)	1.53 (1.13,2.00)

TSH, FT4, FT3, TT4, and TT3 are presented as the median [IQ range, average and 2.5th to 97.5th percentiles (P2.5–P97.5)].

TSH, thyroid stimulating hormone; FT4, free thyroxine; FT3, free triiodothyronine; BMI, body mass index; SBP, systolic blood pressure: TT4, total thyroxine; TT3, total triiodothyronine.

## Discussion

In this study, we collected data on all healthy physical examination populations from the His information system of the Second Affiliated Zhejiang University from 2019 to 2020. The study comprised 6021participants in total removing those who failed to meet the eligibility criteria. We used the American Society of Clinical Chemistry’s guidelines as our criteria for exclusion, and we excluded people who had abnormal liver function. At the same time, we reviewed the indoor quality control and interroom quality evaluation of the immunological laboratory of the laboratory department of our hospital from 2019 to 2020 and believed that these records were completely under control. To characterize the RIs of thyroid-related hormones, we analyzed information gathered from P2.5 and P97.5.

The levels of TSH, FT3, FT4, and TT4 were substantially distinct in males and females, and changes in TSH increased with age. This conclusion is consistent with existing reports (13). Furthermore, TSH in Italians adults 0-105 years old showed an unfavorable linear correlation with age (14). These research discrepancies from ours may be a consequence of using various methods or ethnic backgrounds. We also found that in this study, there were large differences in TT4 and FT3 between males and females (p<0.01). This may be related to body fat rate and estrogen levels in both males and females (13). In our study, the TSH levels of males and females aged ≥50 years were 1.42 (0.51, 4.03) and 1.72 (0.63, 4.31), respectively. In existing reports (15), the RIS of TSH in Chinese elderly individuals was 2.28 (0.35, 8.79). The variations observed among these studies possibly originate from differential exclusion standards and demographic attributes. In this study, we excluded people with abnormal liver function and used people with relatively good nutritional levels. It is worth noting that if people with abnormal liver function are not excluded, the calculated RIS is basically consistent with the reported RIS. Therefore, these existing problems need to be further analyzed in future research.

Although previous studies have established the RIs of thyroid-related hormones, this study has its own advantages: 1. The adopted indirect method is more convenient and economical and does not require volunteer recruitment or additional testing. 2. We collected data from the second hospital affiliated Zhejiang university who performed health examinations in the past two years. The samples all used the Abbott immunoassay method to avoid differences in results caused by different methodologies. 3. According to strict exclusion criteria, complete examination information, including thyroid ultrasound results and surgical palpation results, was obtained for all included individuals. Consequently, the specified RIs might offer higher accuracy and relevance for clinical labs using the same approach. 4. It is worth noting that in this study, we excluded people with abnormal liver function when selecting normal people. It is reported that patients with fatty liver, cirrhosis and other liver diseases, their thyroid function will have varying degrees of decline, thyroid related hormones will also have great changes. (16, 17) This study has many limitations. The immune system responses determined here are best suited to the Chinese coastal populace when examined with the Abbott immunoassay analyzer, due to substantial variations among immune detection methods. Several differences in thyroid-related levels of hormones are being documented among different ethnic groups, which is intriguing (18–25). The main population in Zhejiang Province is Han, and the population included in this study needs to be further subdivided in terms of ethnicity. In addition, although we can assume that Zhejiang Province is a coastal province and that the iodine intake of the resident population should be adequate, the iodine status related to thyroid disease needs to be further evaluated.

A global instance of acute hyperthyroidism is now recognized as aTSH level at the RIs’ threshold combined with Free Thyroxine (FT4) and Free Triiodothyronine (FT3) indices above the maximum value of the RIS. Moderate hyperthyroidism is indicated by TSH values above the maximum range and FT4 levels at the minimal range of the RIS. Subclinical thyroid dysfunction is indicated by TSH levels over the threshold and FT4 levels within the reference interval range. Subclinical hyperthyroidism is recognized when blood TSH levels are below the lowered threshold and FT3 and FT4 levels are within the normal range (26, 27). The establishment of a thyroid-stimulated hormone identification system in China’s coastal cities is significant, as it can help prevent those with minor spikes or declines from being misclassified. It is worth noting that the diagnosis of thyroid disease is not just about hormone detection numbers but should also be combined with clinical manifestations to diagnose and treat thyroid dysfunction (28).

In conclusion, Our study emphasizes the significance of determining thyroid-related hormonal RISs that are age-related and gender-specific in China’s coastal metropolitan population based on the methodology of the second hospital affiliated with Zhejiang University. In our postprocessing work, we need to pay more attention to laboratory external quality assessment (EQA) and indoor quality control (IQC) to make the testing work more detailed and accurate, thus reducing the deviation and coefficient of variation between different testing systems.

## Data Availability

The raw data supporting the conclusions of this article will be made available by the authors, without undue reservation.
